# Trends in Mortalities due to Sudden Cardiac Arrest in the United States Population

**DOI:** 10.1002/joa3.70240

**Published:** 2025-12-02

**Authors:** Muhammad Shaheer Bin Faheem, Tehreem Asghar, Faheem Feroze, Fatima Naveed, Natasha Chowdhury, Sumaya Samadi, Muhammad Aamir Laghari, Jamal S. Rana, Marat Fudim

**Affiliations:** ^1^ Karachi Institute of Medical Sciences, KIMS Karachi Pakistan; ^2^ Akhtar Saeed Medical and Dental College Lahore Pakistan; ^3^ Combined Military Hospital (CMH) Rawalpindi Rawalpindi Pakistan; ^4^ Rawal Institute of Health Sciences Islamabad Pakistan; ^5^ West Virginia School of Osteopathic Medicine Lewisburg West Virginia USA; ^6^ Kabul University of Medical Sciences “Abu Ali Ibn Sina” Kabul Afghanistan; ^7^ Department of Cardiology Lehigh Valley Health Network Allentown Pennsylvania USA; ^8^ Department of Cardiology The Permanente Medical Group Oakland California USA; ^9^ Duke University Medical Center Durham North Carolina USA

**Keywords:** CDC WONDER, sudden cardiac arrest, United States

## Abstract

**Background:**

Sudden cardiac arrest is a leading cause of cardiovascular mortality, often occurring without warning and resulting in high fatality rates. Despite advances in prevention and emergency response, disparities in mortality remain rampant across demographic and geographic groups. This study examines long‐term trends in sudden cardiac arrest‐related mortality in the United States from 1999 to 2023, stratified by sex, race/ethnicity, region, and urbanization level.

**Methods:**

Mortality data was extracted from the CDC WONDER database. Age‐adjusted mortality rates (AAMRs) were calculated per 100 000 persons using the 2000 U.S. standard population. Joinpoint regression analysis was applied to estimate annual percent changes (APCs) and identify significant shifts in mortality trends.

**Results:**

A total of 8 523 980 SCA deaths were reported during the study period. Overall, the AAMR declined from 196.03 in 1999 to 131.55 in 2023, with the most marked reductions observed after 2021 (APC: –9.63; 95% CI: −13.51 to −5.20; *p* < 0.000001). Men consistently exhibited higher AAMRs than women (156.08 vs. 111.09 in 2023). Non‐Hispanic Black individuals had the highest mortality rates (235.18), followed by Hispanics (184.38). Geographic disparities were evident, with the Northeast and metropolitan areas reporting the greatest AAMRs.

**Conclusions:**

Mortality due to sudden cardiac arrest has declined substantially over the past 25 years, likely driven by improvements in cardiovascular prevention, acute care, and resuscitation practices. However, significant sex, racial, and regional disparities persist, highlighting the need for interventions that are tailored to reduce inequities and improve survival from sudden cardiac arrest.

## Graphical abstract

From 1999 to 2023, over 8.5 million U.S. deaths were due to sudden cardiac arrest. The age‐adjusted mortality rate declined from 196.0 to 131.6 per 100 000, with marked reductions after 2021. Mortality remained higher in males, African Americans, and metropolitan regions.

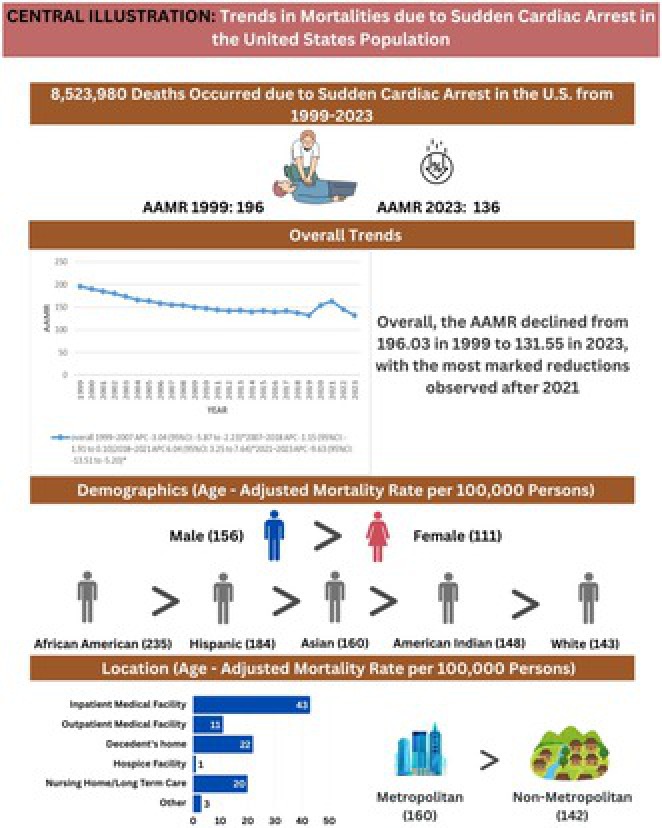

## 1 Introduction

Cardiac arrest is a sudden and devastating cardiovascular occurrence, often leading to death within minutes without prompt intervention. Out‐of‐hospital cardiac arrest happens approximately 356 000 times annually in the United States, and the majority of them are fatal, with previous survival rates being below 10% [1]. While most literature and policy discussions focus on emergency response and post‐resuscitation, there is growing recognition of sudden cardiac arrest (SCA)—events that occur before the average life expectancy—as a distinct public health concern.

Sudden death from cardiac arrest imposes not only severe emotional burdens on families but also measurable losses in workforce productivity and national health costs [2]. Despite public health campaign efforts targeting risk factors such as hypertension, obesity, smoking, and sedentary lifestyle, cardiac arrest continues to disproportionately affect certain demographic groups and regions. Non‐Hispanic Black Americans, for instance, have far greater rates of sudden cardiac death compared with White counterparts, largely due to comorbidity burdens, slower access to care, and systemic disparities [3, 4]. A persistent “Southern disadvantage” also contributes to higher cardiovascular mortality in the southeastern United States above other regions [5]. Modifiable individual‐level risk factors and broader social determinants, such as healthcare access, income inequality, and education, drive these disparities [6], which are then further exacerbated in urban settings, with high rates of diabetes and metabolic syndrome continuing to drive cardiac arrest events [7].

Although the Centers for Disease Control and Prevention (CDC) provides extensive data on cardiovascular mortality, scientific research on sudden cardiac arrest, particularly age‐adjusted analyses, remains limited. National systems often group cardiac arrest with broader cardiovascular mortality or lack age‐disaggregated data, leaving gaps in long‐term trends and demographic variations.

This study addresses that gap by using 25 years of nationally representative mortality data from the CDC WONDER database from 1999 to 2023. We present a comprehensive analysis of sudden cardiac arrest mortality among U.S. adults by gender, race/ethnicity, region, and urbanization status. By calculating age‐adjusted mortality rates (AAMRs) and annual percentage changes (APCs), this study highlights temporal trends and structural disparities. These findings aim to inform policymakers, clinicians, and public health practitioners about where the burden of SCA lies and where prevention efforts should be prioritized.

## 2 Methods

### 2.1 Study Setting and Population

In this descriptive, population‐based study, we analyzed national mortality data from the Centers for Disease Control and Prevention Wide‐ranging Online Data for Epidemiologic Research (CDC WONDER) platform [8]. Data were obtained from the Multiple Cause of Death files spanning from 1999 to 2023. These files are compiled from U.S. death certificates submitted by all 50 states and the District of Columbia, and have been extensively used in prior studies to examine trends in cardiovascular mortality.

Premature cardiac arrest‐related mortality was defined as deaths due to cardiac arrest (ICD‐10 code I46.9) occurring in adults aged 25 to 64 years, consistent with previous studies that characterize sudden cardiac mortality as death occurring before the age of 65 years [9]. Deaths outside this age range were excluded from the analysis to focus specifically on early, potentially preventable cardiac mortality.

### 2.2 Data Abstraction

We extracted data on year of death, age, sex, race/ethnicity, urbanization level, geographic region, and place of death. Race and ethnicity were classified into five mutually exclusive groups based on CDC WONDER standards: non‐Hispanic (NH) White, NH Black or African American, NH Asian or Pacific Islander, NH American Indian or Alaska Native, and Hispanic or Latino. Place of death was categorized as inpatient medical facilities, outpatient/emergency departments, nursing homes/long‐term care facilities, hospice, home, or other/unknown. Urbanization level was determined using the 2013 NCHS Urban–Rural Classification Scheme for counties [10]. Geographic regions were defined according to the U.S. Census Bureau classification: Northeast, Midwest, South, and West [11].

### 2.3 Statistical Analysis

We calculated age‐adjusted mortality rates (AAMRs) per 100 000 persons using the direct method, standardized to the 2000 U.S. standard population [12]. Crude mortality rates were also calculated for descriptive reporting. Rates were stratified by demographic and geographic characteristics.

To assess temporal trends, we used Joinpoint regression analysis (Joinpoint Regression Program v5.0.2, National Cancer Institute) to determine annual percent change (APC) and identify statistically significant changes in trend over time. This method fits log‐linear regression models to mortality data and uses permutation tests to identify years when significant shifts in trend occurred [13]. An APC was considered significant if the slope of the trend line differed from zero at *p* < 0.05.

### 2.4 Ethical Considerations

This study used publicly available, de‐identified data and was therefore exempt from institutional review board approval. All methods complied with the STROBE (Strengthening the Reporting of Observational Studies in Epidemiology) guidelines for reporting observational studies.

## 3 Results

### 3.1 Proportional Mortality Rate Across Different Variables From 1999 to 2023

Total 8 523 980 deaths were reported from sudden cardiac arrest, showing 49.4% of males and 50.6% of females. Mortalities were highest in NH Whites (73.18%) followed by NH African American (13.83%), Hispanic or Latino (8.76%), NH Asian (3.7%) and lastly NH American Indians (0.53%). A large number of deaths occurred in in‐patient medical facilities (43.23%) with lesser frequencies in decedent's (22.12%), nursing homes (19.66%), outpatient (10.98%) and other facilities (3.21%) while least (0.8%) recorded from hospice care. Regionally deaths were higher in metropolitan areas (84.09%) than in non‐metropolitan areas (15.91%). The South region accounted for the highest number of deaths (31.94%) as compared to the Northeast (27.88%), West (27.59%) and Midwest (12.59%) regions respectively (Table S1).

### 3.2 Overall Age‐Adjusted Trends for Sudden Cardiac Arrest‐Related Mortality in Adults From 1999 to 2023

**FIGURE 1:**
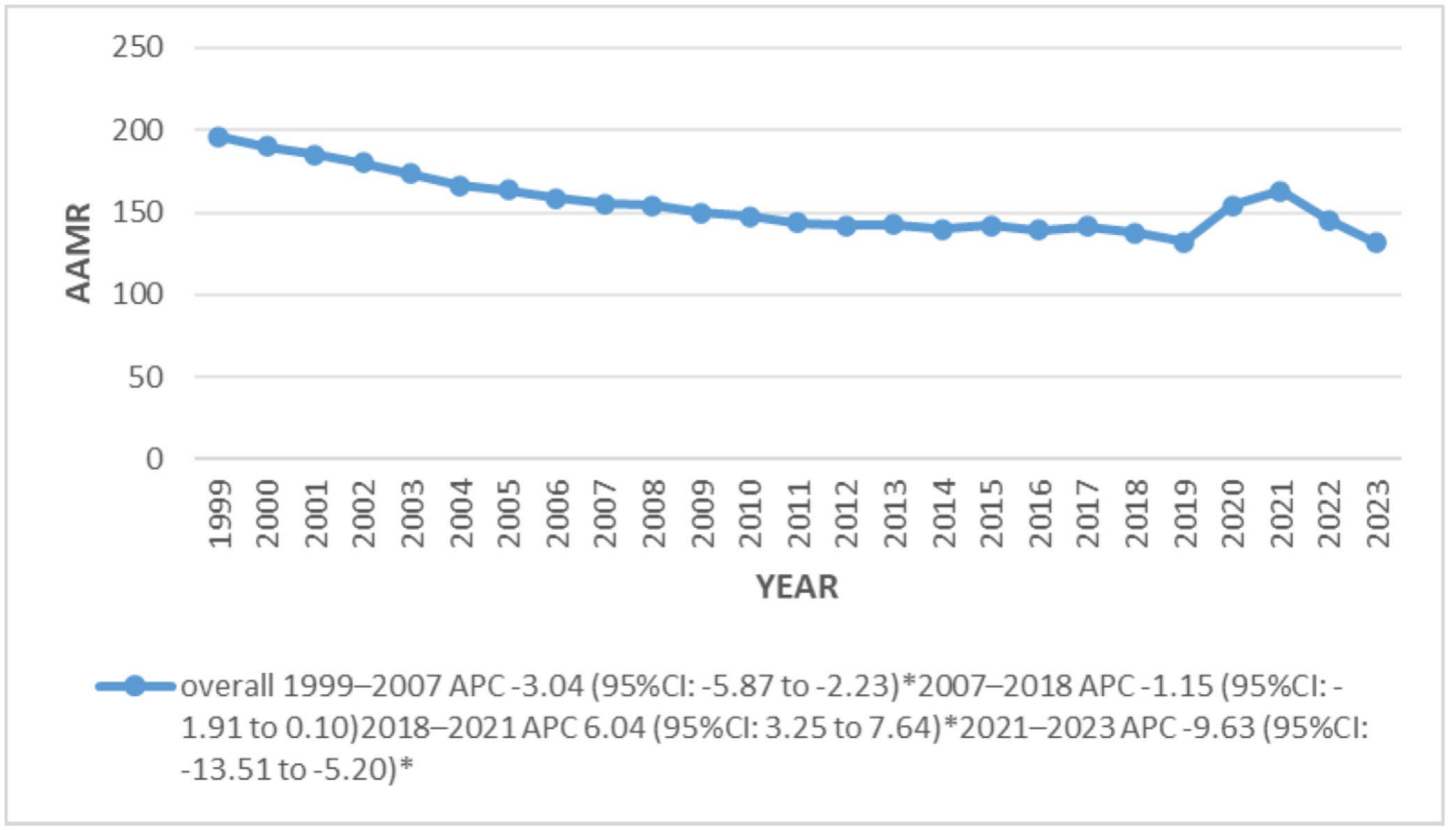
Overall, trends in sudden cardiac arrest‐related age‐adjusted mortality rates per 100 000 among adults aged 25 years and above in the United States, 1999 to 2023. *Note*: * correspond that values to in figure representation.

The AAMR decreased 1.5 folds from 196.03 in 1999 to 131.55 in 2023. Significant declining trends were observed in AAMRs throughout the study period with a more prominent decline noted from 2021 to 2023 (APC: −9.63*; 95% CI: −13.51 to −5.20; *p* < 0.000001) (Figure 1) (Tables S2 and S3).

### 3.3 Demographic Trends

Notable variances were shown by AAMRs across different demographic subgroups.

#### 3.3.1 Gender Stratified

**FIGURE 2:**
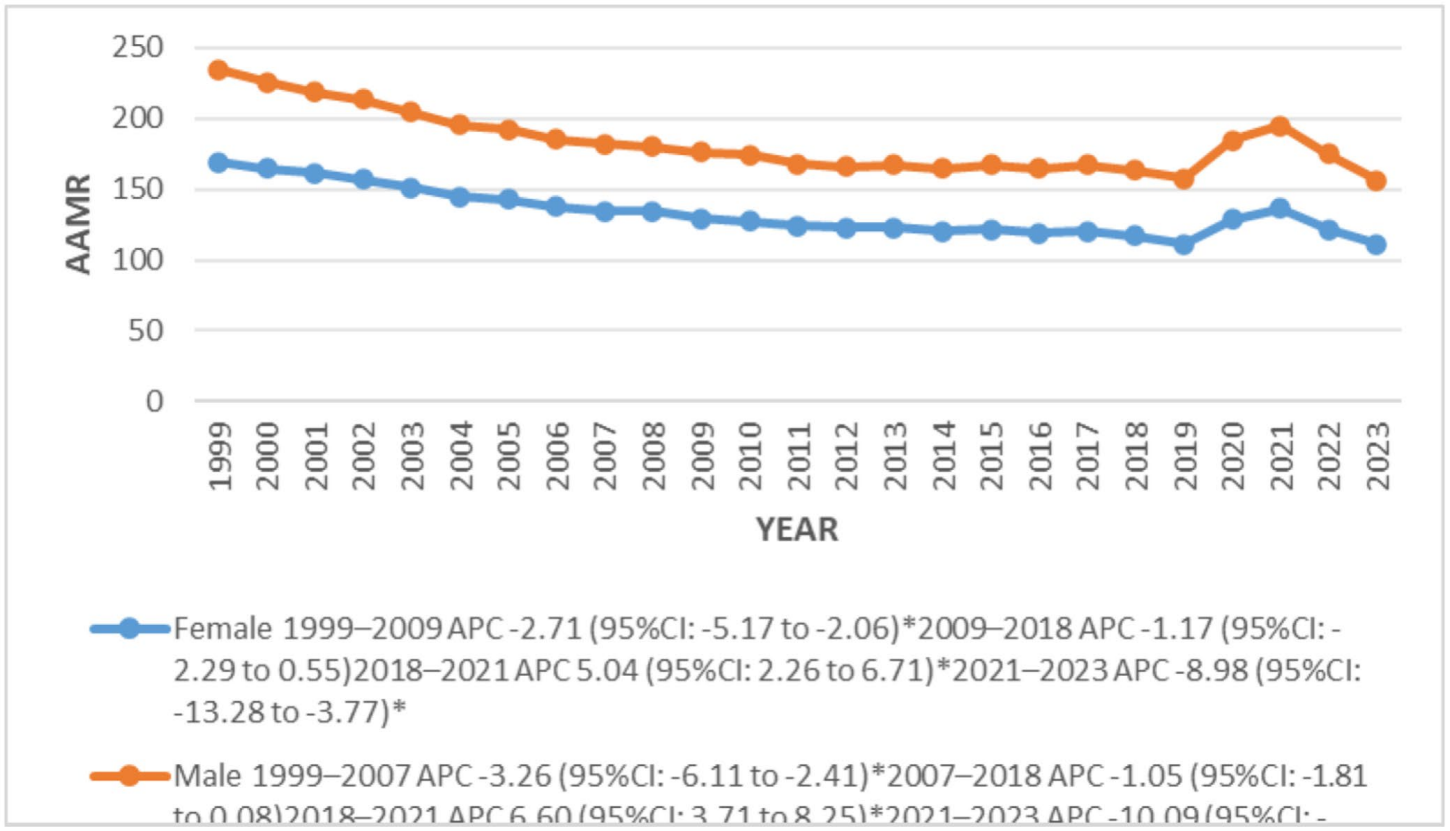
Trends in sudden cardiac arrest‐related age‐adjusted mortality rates per 100 000, stratified by sex among adults aged 25 years and above in the United States, 1999 to 2023.

The total AAMR reported by 2023 was156.08 in males 1.4 times that of females at 111.09. AAMR showed declining trends during our study in both genders, the trends being significant from 2021 to 2023 in males and females with associated APCs of −10.09 (95% CI: −13.94 to −6.19; *p <* 0.000001) and −8.98 (95% CI: −13.28 to −3.77; *p* = 0.0016) respectively (Figure 2) (Tables S2 and S3).

#### 3.3.2 Race/Ethnicity Stratified

**FIGURE 3:**
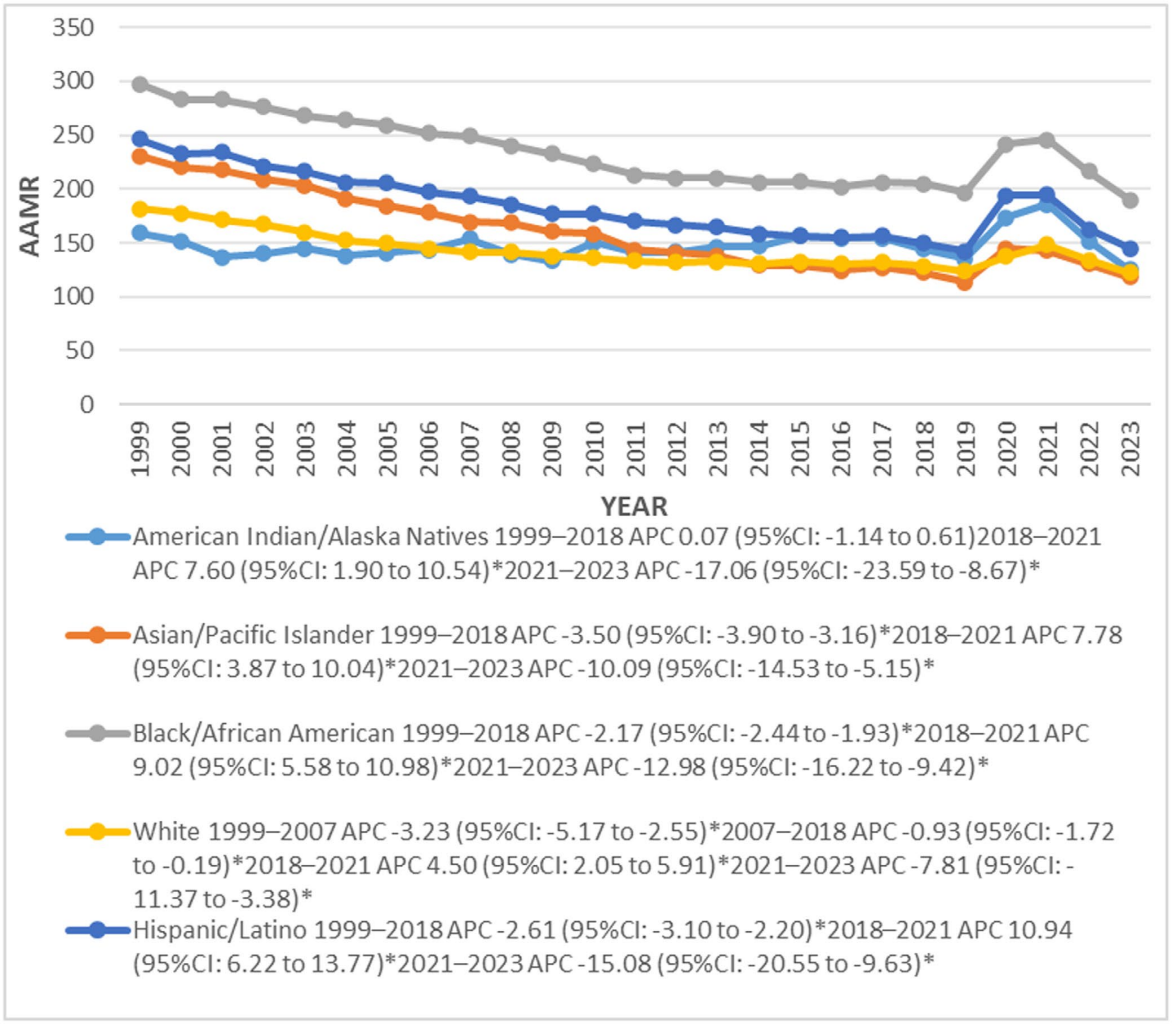
Trends in sudden cardiac arrest‐related age‐adjusted mortality rates per 100 000, stratified by race and ethnicity among adults aged 25 years and above in the United States, 1999 to 2023.

The recorded mortality rates were highest in NH African Americans (235.18) followed by Hispanics (184.38), NH Asians or Pacific Islanders (159.91), NH American Indian or Alaska Native (147.50) and lastly NH White (143.22). From 1999 the AAMR declined significantly up to 2015 in NH Whites (APC *p* = 0.002: 1999–2007: −3.23; 95% CI: −5.17 to −2.55; 2007–2018: −0.93; 95% CI: −1.72 to −0.19) and until 2017 in NH Asians or Pacific Islanders and NH African Americans with associated APCs of −3.50* (95% CI: −3.90 to −3.16; *p* = 0.002) and −2.17* (95% CI: −2.44 to −1.93; *p* < 0.000001) respectively. However, in Hispanics the AAMR declined sharply throughout the study period and from 1999–2018 APC: −2.61* (95% CI: −3.10 to −2.20; *p* = 0.0004) (Figure 3).

### 3.4 Geographic Trends

**FIGURE 4:**
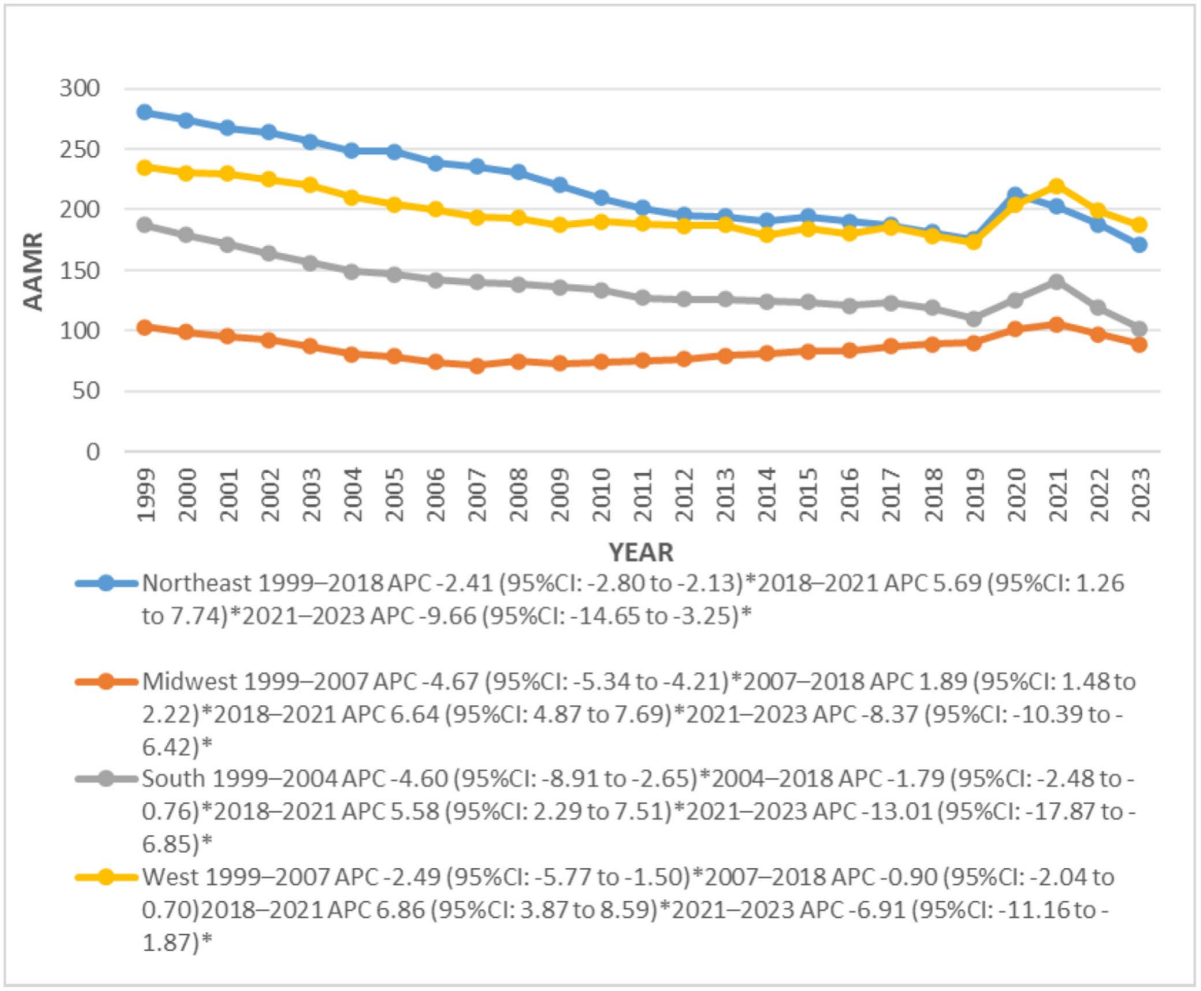
Trends in sudden cardiac arrest‐related age‐adjusted mortality rates per 100 000, stratified by census region among adults aged 25 years and above in the United States, 1999 to 2023.

**FIGURE 5:**
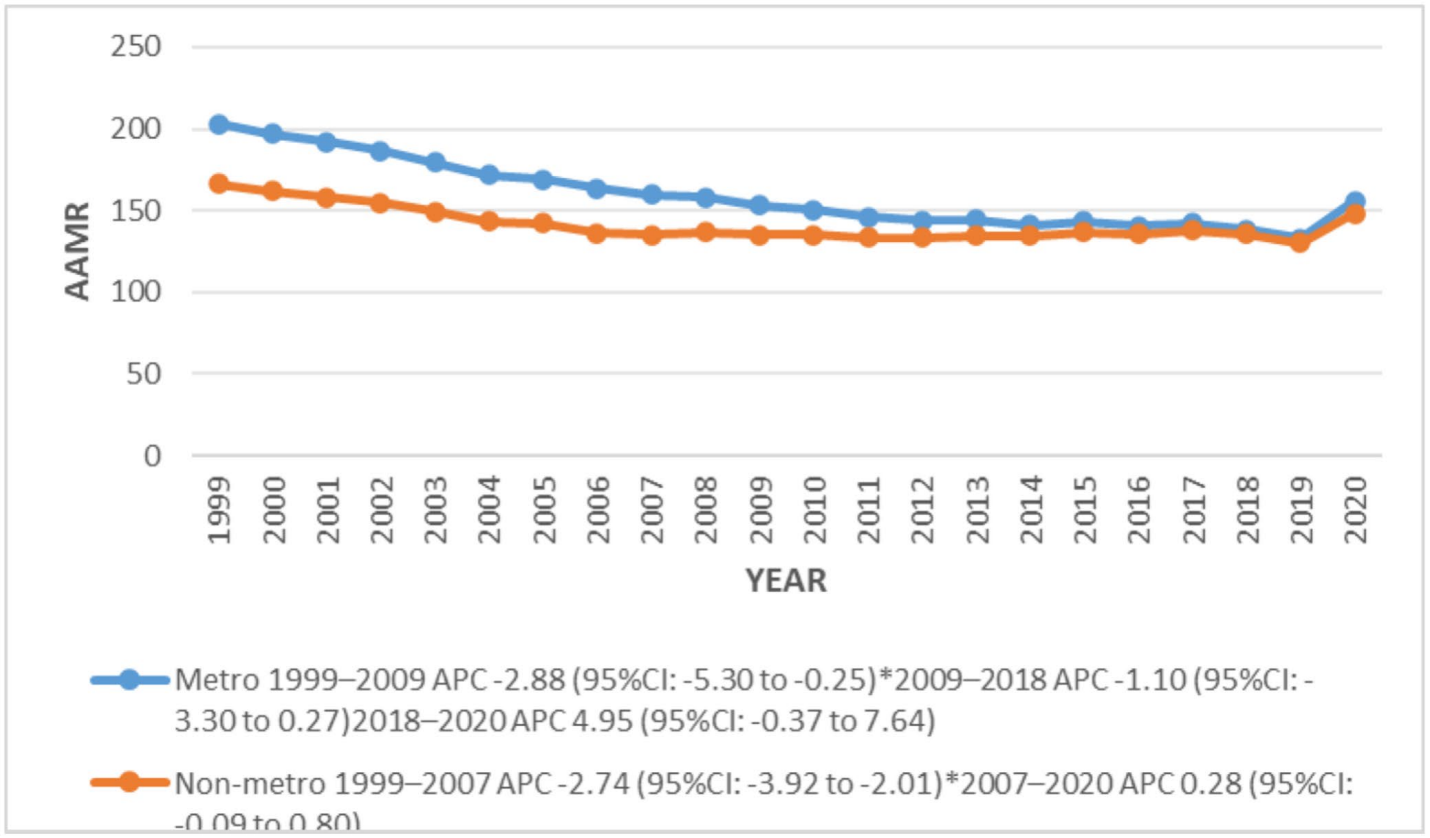
Trends in sudden cardiac arrest‐related age‐adjusted mortality rates per 100 000, stratified by urbanization among adults aged 25 years and above in the United States, 1999 to 2020.

Significant disparities were found among geographical regions and states in the top 90th percentile: New York (384.8), Georgia (293.7), Mississippi (293.4), California (285.7) and Nevada (272.7) had nearly 7‐folds the AAMRs of states Minnesota (35.3), Maryland (36.3), Wisconsin (36.9), Illinois (59.6) and Vermont (57.3) in the lower 10th percentile. Further, AAMR was reported highest from the northeast region (218.3), followed by the South (198.9), West (137.3) and Midwest (85.8) regions, respectively (Figure 4). Regionally higher AAMRs were shown by metropolitan areas (159.6) compared to non‐metropolitan areas (141.6). However, AAMR decreased from 1999 to 2023 in both metropolitan and non‐metropolitan areas, declining significantly from 1999 in metropolitan areas and 2003 in non‐metropolitan areas until 2007 with APCs of −2.9 (95% CI: −5.30 to −0.25; *p* = 0.045991) and −2.7 (95% CI: −3.92 to −2.01; *p* < 0.000001) respectively (Figure 5).

## 4 Discussion

This 25‐year analysis of mortality data from the CDC identifies several key findings. First, overall mortality rates from sudden cardiac arrest (SCA) declined during the study period, with a more prominent decline noted from 2021 to 2023. Second, men were found to have a higher AAMR than women. Third, NH Blacks exhibited the highest AAMR among racial groups. Nonmetropolitan areas were associated with higher AAMRs for mortality due to sudden cardiac arrest (SCA), as was the Northeast region, followed by the South, West, and Midwest regions, respectively, with metropolitan areas also showing relatively high rates. These findings highlight critical disparities that have implications for both disease management guidelines and health policy.

The decline in SCA mortality, especially after 2021, is largely explained by fewer STEMI cases [14]. STEMI carries a higher scar burden and increased mortality. Timely interventions in AMI management are crucial, as myocardial scars often provide substrates for ventricular arrhythmias that lead to sudden cardiac arrest. The decline in AMI cases is mainly attributed to better preventive measures, including greater statin use and risk factor modification. Other causes of SCA, such as channelopathies and non‐ischemic cardiomyopathies, are unlikely to have decreased. Improvements in the survival chain, like high‐quality bystander CPR, may have also contributed to reduced mortality. In addition, updated American Heart Association (AHA) resuscitation guidelines have standardized advanced cardiovascular life support (ACLS) and emphasized optimal management of in‐hospital cardiac arrest, leading to better post‐resuscitation treatments, including therapeutic hypothermia, early percutaneous coronary intervention, and advanced critical care [15]. The initial increase in AAMR from 2019 to 2021 may be attributed to the COVID‐19 pandemic. During this period, the rise in sudden cardiac death (SCD) indicated a bidirectional relationship between COVID‐19 and cardiovascular conditions. Autopsy reports of patients who experienced SCD revealed the presence of the SARS‐CoV virus in the heart tissue, suggesting that inflammation may have contributed to their deaths [16, 17]. Additionally, COVID‐19 infection has been shown to nearly triple the risk of mortality from cardiovascular disease (CVD), highlighting the need for routine monitoring of cardiovascular parameters in affected patients [16].

Our analysis found that SCA‐related AAMRs were slightly higher in men than in women, consistent with earlier studies showing a higher mortality rate among male patients [18]. Zhang et al.'s [18] study found that globally, the premature cardiovascular death rates were nearly 35.6% higher in men, with only around 5% of WHO member states (eight countries in 2016, including Ghana, Mali, Sao Tome and Principe, Zimbabwe, Bhutan, Republic of the Congo, and Nigeria) reporting higher rates in women [18]. However, our study's finding is contradicting Jie et al.'s [19] study, which reported a greater life expectancy (LE) loss from CVD in women than men in China [19]. This gender paradox, where women experience better survival rates despite adverse prognostic factors, suggests differences in SCA pathophysiology between the two sexes. Higher mortality rates in men are linked to a greater prevalence of smoking, alcoholism, and substance abuse, whereas women may benefit from estrogen's protective effects on coronary artery health, contributing to relatively better outcomes in the face of similar medical challenges [20, 21].

Our data highlights significant racial and ethnic disparities among patients, with Non‐Hispanic Black individuals experiencing the highest age‐adjusted mortality rates (AAMRs). The higher SCA mortality rate observed in Black patients compared to White patients is unsurprising, explained by the higher prevalence of hypertension among Black patients [22]. Previous surveillance studies observed in cities such as Seattle, Chicago, New York City, Illinois, and Portland indicate that Black individuals face a greater risk of out‐of‐hospital sudden cardiac arrest [23, 24]. Additionally, cardiac and resuscitation registry data indicate Black patients were less likely to survive in‐hospital sudden cardiac arrests, though the survival gaps have decreased over the past 5 years [23, 24, 25, 26, 27]. Furthermore, LIFE study (Losartan Intervention For Endpoint Reduction in Hypertension) followed 533 black and 8660 non‐Black patients with hypertension over 4.8 years (17 SCD events in Blacks and 161 in non‐Blacks), and found that Black race was associated with almost double the risk of SCA compared with non‐Blacks even after multivariate adjustment (hazard ratio, 1.98; 95% CI, 1.12–3.59) [28]. Research suggests that various factors may contribute to disparities in witnessed cardiac arrests, bystander CPR initiation, and the presence of initial shockable rhythms among different communities. These factors can influence hospital admission rates and outcomes related to survival. It is important to continue addressing these disparities to improve overall health equity and outcomes for all individuals. Survival likelihood increases for witnessed pulmonary cardiac arrests (SCA) due to early recognition and bystander CPR [29]. Therefore, controlling hypertension is crucial, as it is more prevalent and strongly linked to SCA in Black individuals compared with Whites. Despite improvements in hypertension awareness [30] and treatment over the past 30 years, more targeted efforts are needed to enhance hypertension control among Black individuals.

We observed considerable regional differences in AAMRs. The highest rates were reported in the Northeast, followed by the South, West, and Midwest, respectively. Regionally higher AAMRs were shown by metropolitan areas compared to non‐metropolitan areas. Regions with high population density, such as the Northeast and West, may have higher rates of sudden cardiac arrest with a medical response due to factors including longer response times, delays in CPR initiation, lack of early defibrillation, and extended transport times to hospitals [31]. Cardiovascular risk factors, such as obesity, smoking, diet, diabetes, and substance use, vary significantly across the United States, potentially leading to cases of SCA. Critical gaps in SCA care remain, including limited resources related to geography or population density, baseline differences in cardiovascular risk, and disparities in CPR training. Lower CPR training rates at the county level might contribute to decreased rates of bystander CPR, fewer hospital survival cases, and higher SCA mortality rates. Community‐level initiatives that promote early recognition of sudden cardiac arrest and widespread CPR training can enhance SCA outcomes across regions.

## 5 Limitations

The CDC WONDER database, while extensive, has limitations that affect our study. It relies on the accuracy of data from death certificates and includes only information from death reports, excluding details such as comorbidities, medical history, medications, vital signs, laboratory results, and CPR or AED usage. Furthermore, it does not provide information on whether CPR was given or any adverse drug effects that patients experienced. Including CPR and AED usage data would allow for a more nuanced analysis of the factors that influence survival rates and recovery in emergencies. This addition would likely enhance the validity of findings and contribute to better‐informed clinical guidelines and public health strategies. Additionally, causes of death are captured using ICD coding, which can have errors. Our retrospective cross‐sectional analysis cannot establish causal relationships between county‐level characteristics and mortality. We focused on overall estimates of the age‐adjusted mortality rate (AAMR) rather than individual risk factors, as the database provides only aggregated data, which restricts our ability to perform adjusted analyses. Further research is needed in this domain to address these gaps.

## 6 Conclusion

This 25‐year CDC‐based analysis demonstrates a consistent decline in age‐adjusted mortality rates (AAMRs) from sudden cardiac arrest (SCA), notably after 2021, driven by reductions in STEMI incidence, improved prevention, and advancements in cardiac care. Men showed higher AAMRs than women, reflecting greater exposure to risk factors. Non‐Hispanic Black individuals experienced the highest AAMRs, largely due to hypertension prevalence and disparities in emergency response and care. Regionally, the Northeast and metropolitan areas reported elevated mortality, potentially due to delayed EMS response and lower CPR training rates. These findings underscore the need for targeted interventions and equitable public health strategies to further reduce SCA‐related mortality.

## Author Contributions

Author Muhammad Shaheer Bin Faheem: contributed to conceptualization, writing – original draft, writing – review and editing, data curation, visualization, formal analysis, software, and project administration. Authors Tehreem Asghar and Faheem Feroze: contributed to validation, writing – original draft, data curation, and visualization. Author Fatima Naveed: contributed to methodology and writing – original draft. Authors Natasha Chowdhury and Sumaya Samadi: contributed to investigation, resources, and writing – original draft. Authors Muhammad Aamir Laghari, Jamal S. Rana and Marat Fudim: contributed to writing – review and editing, and supervision.

## Funding

The authors has nothing to report.

## Ethics Statement

The present study does not involve direct data collection from human subjects or animals. All data used in this study were obtained from previously published and publicly available sources, adhering to the ethical guidelines of the respective studies. No identifiable patient information is presented in this study.

## Conflicts of Interest

The authors declare no conflicts of interest.

## Supporting information


**Data S1:** joa370240‐sup‐0001‐DataS1.docx.

## Data Availability

The datasets generated and/or analyzed during the current study are available in the manuscript or as Supporting Information.
