# Supplementary material for: Trends in Mortalities due to Sudden Cardiac Arrest in the United States Population
Source: J Arrhythm. 2025 Dec 2;41(6):e70240. doi: 10.1002/joa3.70240 (PMC12670185; doi:10.1002/joa3.70240)
Supplement: Supplementary file 1 — Data S1: joa370240‐sup‐0001‐DataS1.docx. [file JOA3-41-e70240-s001.docx]

| **Variables** | **Deaths** | **% of Total Deaths** |
| --- | --- | --- |
| Overall | 8523980 | 100% |
| **Gender** | | |
| Female | 4317560 | 50.6% |
| Male | 4206420 | 49.4% |
| **Race** | | |
| NH White | 6238252 | 73.18% |
| NH Black or African American | 1178709 | 13.83% |
| NH Asian or Pacific Islander | 315360 | 3.7% |
| NH American Indian or Alaskan Native | 44894 | 0.53% |
| Hispanic or Latino | 746765 | 8.76% |
| **Age Groups** | | |
| 25-34 | 62446 | 0.74% |
| 35-44 | 170787 | 2.00% |
| 45-54 years | 489190 | 5.74% |
| 55-64 years | 1057679 | 12.41% |
| 65-74 years | 1663065 | 19.51% |
| 75-84 years | 2371502 | 27.82% |
| 85+ years | 2709311 | 31.78% |
| **Place of Deaths** | | |
| Medical Facility - Inpatient | 3604414 | 43.23% |
| Medical Facility - Outpatient or ER | 915722 | 10.98% |
| Medical Facility - Dead on Arrival | 66734 | 0.8% |
| Decedent's home | 1844370 | 22.12% |
| Nursing home/long term care | 1639134 | 19.66% |
| Other | 268341 | 3.21% |
| **Urbanization** | | |
| Metropolitan Areas | 6187769 | 84.09% |
| Non-Metropolitan Areas | 1170475 | 15.91% |
| **Regions** | | |
| Northeast | 2376587 | 27.88% |
| Midwest | 1073365 | 12.59% |
| South | 2722541 | 31.94% |
| West | 2351487 | 27.59% |
| **NH: Non-Hispanic** | | |

**Supplemental Table 1.** Absolute number of sudden cardiac arrest-related deaths and total deaths percentages among adults aged 25 and above stratified by overall, gender, race/ ethnicity, age group, place of death, urbanization and regions in the United States, 1999-2023.

**Supplementary Figure 1.** Trends in sudden cardiac arrest related age-adjusted mortality rates, stratified by age groups in the United States, 1999 to 2023.

APC = Annual Percentage Change, CI = Confidence Interval.

*Indicates that the Annual Percentage Change (APC) is significantly different from zero at α = 0.05.

| **Age-Adjusted Rate Per 100,000 (95% CI)** | | | |
| --- | --- | --- | --- |
| **Year** | **Overall** | **Male** | **Female** |
| 1999 | 196.03  (195.38 - 196.69) | 234.59  (233.42 - 235.76) | 168.93  (168.16 - 169.71) |
| 2000 | 190.06  (189.42 - 190.70) | 226.00  (224.86 - 227.14) | 164.70  (163.93 - 165.46) |
| 2001 | 185.22  (184.59 - 185.85) | 218.69  (217.58 - 219.80) | 161.50  (160.75 - 162.25) |
| 2002 | 180.32  (179.71 - 180.94) | 213.52  (212.44 - 214.60) | 156.72  (155.99 - 157.46) |
| 2003 | 173.62  (173.02 - 174.21) | 204.42  (203.38 - 205.47) | 151.32  (150.61 - 152.04) |
| 2004 | 166.34  (165.76 - 166.92) | 195.65  (194.63 - 196.66) | 144.91  (144.22 - 145.61) |
| 2005 | 163.81  (163.24 - 164.38) | 192.31  (191.32 - 193.30) | 142.76  (142.07 - 143.45) |
| 2006 | 158.38  (157.83 - 158.94) | 185.56  (184.60 - 186.51) | 138.03  (137.36 - 138.70) |
| 2007 | 155.10  (154.56 - 155.65) | 182.38  (181.44 - 183.32) | 134.60  (133.94 - 135.26) |
| 2008 | 154.12  (153.58 - 154.66) | 180.18  (179.26 - 181.10) | 134.34  (133.69 - 134.99) |
| 2009 | 149.72  (149.19 - 150.24) | 176.07  (175.18 - 176.97) | 129.57  (128.93 - 130.21) |
| 2010 | 147.53  (147.02 - 148.05) | 174.10  (173.21 - 174.98) | 127.27  (126.65 - 127.90) |
| 2011 | 143.48  (142.97 - 143.98) | 168.01  (167.16 - 168.86) | 124.32  (123.70 - 124.93) |
| 2012 | 141.94  (141.45 - 142.44) | 166.29  (165.46 - 167.13) | 122.89  (122.29 - 123.50) |
| 2013 | 142.53  (142.04 - 143.02) | 167.46  (166.63 - 168.29) | 122.90  (122.30 - 123.50) |
| 2014 | 139.87  (139.38 - 140.35) | 164.70  (163.89 - 165.51) | 120.15  (119.56 - 120.74) |
| 2015 | 141.92  (141.44 - 142.40) | 167.02  (166.22 - 167.82) | 121.81  (121.22 - 122.40) |
| 2016 | 139.43  (138.96 - 139.90) | 164.65  (163.86 - 165.44) | 119.11  (118.54 - 119.69) |
| 2017 | 141.40  (140.93 - 141.87) | 167.24  (166.45 - 168.02) | 120.51  (119.94 - 121.09) |
| 2018 | 137.74  (137.28 - 138.20) | 163.13  (162.37 - 163.89) | 117.06  (116.50 - 117.63) |
| 2019 | 132.32  (131.87 - 132.76) | 157.58  (156.84 - 158.32) | 111.69  (111.14 - 112.23) |
| 2020 | 154.16  (153.68 - 154.64) | 184.86  (184.07 - 185.65) | 128.75  (128.17 - 129.33) |
| 2021 | 162.99  (162.49 - 163.49) | 194.88  (194.05 - 195.71) | 136.30  (135.68 - 136.91) |
| 2022 | 145.41  (144.95 - 145.88) | 174.75  (173.98 - 175.52) | 121.66  (121.10 - 122.23) |
| 2023 | 131.55  (131.11 - 131.99) | 156.08  (155.35 - 156.80) | 111.09  (110.55 - 111.63) |
| **Total** | 155.00 (154.474-155.52) | 183.2048  (181.35-184.10) | 133.3156  (132.68-133.95) |

**Supplemental Table 2.** Overall and sex-stratified sudden cardiac arrest related age-adjusted mortality rates per 100,000 among adults aged 25 and above in the United States, 1999 to 2023.

**Supplementary Figure 2.** Percent sudden cardiac arrest related total deaths by place of deaths among adults aged 25 and above in the United States, 1999 to 2023.

| **Year Interval** | **APC (95% CI)** |
| --- | --- |
| **Overall** | |
| 1999-2007 | -3.04* (−5.87 – −2.23) |
| 2007-2018 | -1.15 (−1.91 – 0.10) |
| 2018-2021 | 6.04* (3.25 – 7.64) |
| 2021-2023 | -9.63* (−13.51 – −5.20) |
| **Gender** | |
| **Male** | |
| 1999-2007 | -3.26* (−6.11 – −2.41) |
| 2007-2018 | -1.05 (−1.81 – 0.08) |
| 2018-2021 | 6.60* (3.71 – 8.25) |
| 2021-2023 | -10.09* (−13.94 – −6.19) |
| **Female** | |
| 1999-2009 | -2.71* (−5.17 – −2.06) |
| 2009-2018 | -1.17 (−2.29 – 0.55) |
| 2018-2021 | 5.04* (2.26 – 6.71) |
| 2021-2023 | -8.98* (−13.28 – −3.77) |
| **Race** | |
| **NH American Indian or Alaska Native** | |
| 1999-2018 | 0.07 (−1.14 – 0.61) |
| 2018-2021 | 7.60* (1.90 – 10.54) |
| 2021-2023 | -17.06* (−23.59 – −8.67) |
| **NH Asian or Pacific Islander** | |
| 1999-2018 | -3.50* (−3.90 – −3.16) |
| 2018-2021 | 7.78* (3.87 – 10.04) |
| 2021-2023 | -10.09* (−14.53 – −5.15) |
| **NH Black or African American** | |
| 1999-2018 | -2.17* (−2.44 – −1.93) |
| 2018-2021 | 9.02* (5.58 – 10.98) |
| 2021-2023 | -12.98* (−16.22 – −9.42) |
| **NH White** | |
| 1999-2007 | -3.23* (−5.17 – −2.55) |
| 2007-2018 | -0.93* (−1.72 – −0.19) |
| 2018-2021 | 4.50* (2.05 – 5.91) |
| 2021-2023 | -7.81* (−11.37 – −3.38) |
| **Hispanic or Latino** | |
| 1999-2018 | -2.61* (−3.10 – −2.20) |
| 2018-2021 | 10.94* (6.22 – 13.77) |
| 2021-2023 | -15.08* (−20.55 – −9.63) |
| **Age Groups** | |
| **Young** | |
| 1999-2018 | 0.00 (−0.62 – 0.48) |
| 2018-2021 | 18.39* (12.00 – 22.21) |
| 2021-2023 | -15.14* (−21.96 – −7.93) |
| **Middle** | |
| 1999-2007 | -2.30* (−5.97 – −1.25) |
| 2007-2018 | 0.50 (−0.39 – 1.80) |
| 2018-2021 | 9.92* (6.19 – 11.93) |
| 2021-2023 | -13.64* (−18.09 – −9.52) |
| **old** | |
| 1999-2007 | -3.24* (−5.87 – −2.49) |
| 2007-2018 | -1.57* (−2.29 – −0.41) |
| 2018-2021 | 4.45* (1.96 – 5.89) |
| 2021-2023 | -8.23* (−11.95 – −3.67) |
| **Urbanization** | |
| **Metropolitan** | |
| 1999-2009 | -2.88* (−5.30 – −0.25) |
| 2009-2018 | -1.10 (−3.30 – 0.27) |
| 2018-2020 | 4.95 (−0.37 – 7.64) |
| **Non-Metropolitan** | |
| 1999-2007 | -2.74* (−3.92 – −2.01) |
| 2007-2020 | 0.28 (−0.09 – 0.80) |
| **Regions** | |
| **Northeast** | |
| 1999-2018 | -2.41* (−2.80 – −2.13) |
| 2018-2021 | 5.69* (1.26 – 7.74) |
| 2021-2023 | -9.66* (−14.65 – −3.25) |
| **Midwest** | |
| 1999-2007 | -4.67* (−5.34 – −4.21) |
| 2007-2018 | 1.89* (1.48 – 2.22) |
| 2018-2021 | 6.64* (4.87 – 7.69) |
| 2021-2023 | -8.37* (−10.39 – −6.42) |
| **South** | |
| 1999-2004 | -4.60* (−8.91 – −2.65) |
| 2004-2018 | -1.79* (−2.48 – −0.76) |
| 2018-2021 | 5.58* (2.29 – 7.51) |
| 2021-2023 | -13.01* (−17.87 – −6.85) |
| **West** | |
| 1999-2007 | -2.49* (−5.77 – −1.50) |
| 2007-2018 | -0.90 (−2.04 – 0.70) |
| 2018-2021 | 6.86* (3.87 – 8.59) |
| 2021-2023 | -6.91* (−11.16 – −1.87) |
| **NH: Non-Hispanic; APC: Annual Percent Change** | |

**Supplemental Table 3.** Annual percent change (APC) of sudden cardiac arrest-related age-adjusted mortality rates per 100,000 among adults aged 25 and above in the United States, 1999 to 2023.


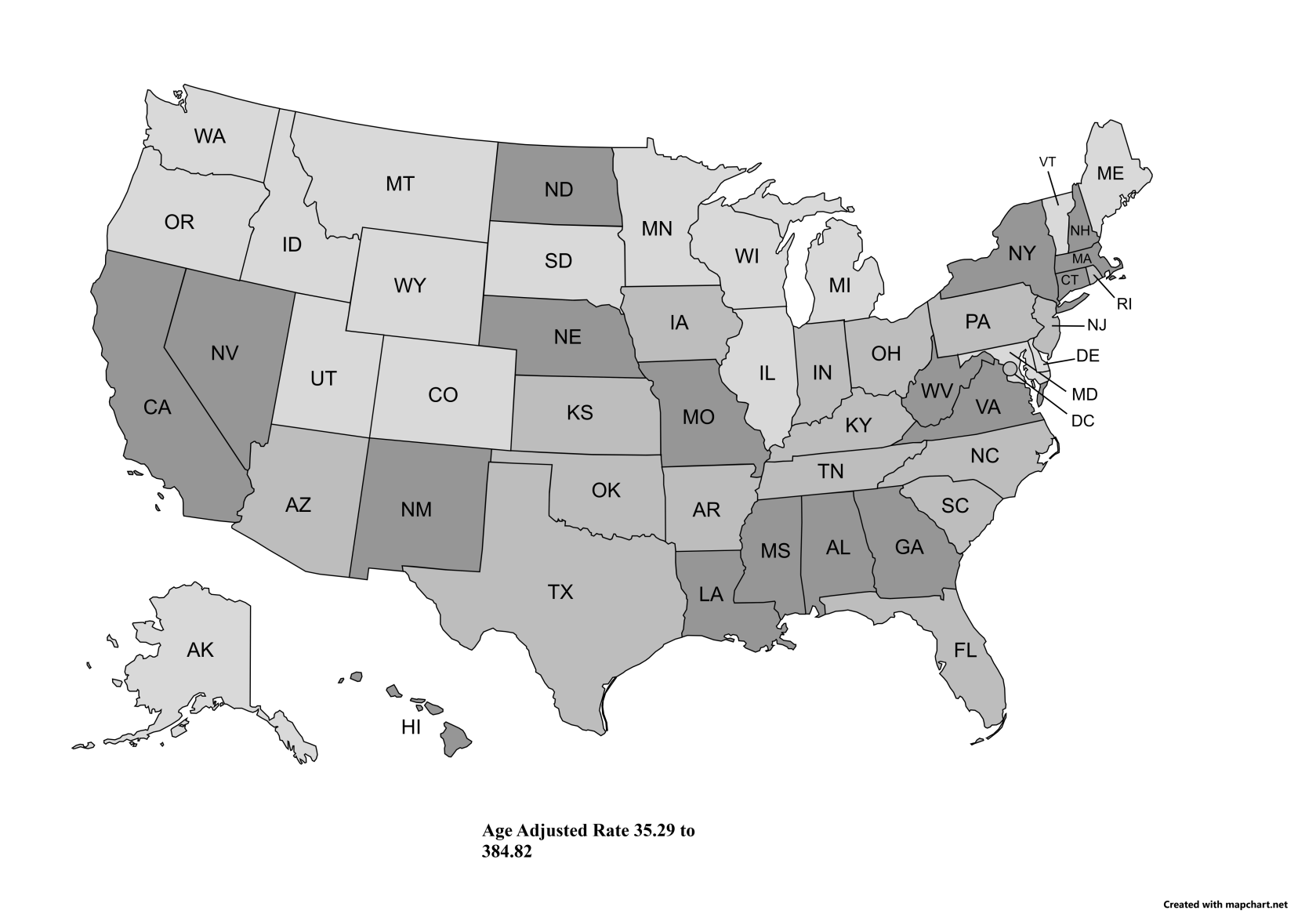
**Supplementary Figure 3.** prematre Cardiac arrest-related age-adjusted mortality rates per 100,000, stratified by states among adults aged 25 and above in the United States, 1999 to 2020.

| **Age-Adjusted Rate Per 100,000 (95% CI)** | | | | | |
| --- | --- | --- | --- | --- | --- |
| **Year** | **NH American Indian or Alaska Native** | **NH Asian or Pacific Islander** | **NH Black or African American** | **NH White** | **Hispanics or Latino** |
| 1999 | 159.49  (149.55 - 169.43) | 231.00  (225.86 - 236.14) | 297.44  (294.64 - 300.25) | 181.94  (181.26 - 182.63) | 246.40  (242.85 - 249.96) |
| 2000 | 151.60  (142.47 - 160.73) | 220.78  (215.94 - 225.61) | 283.27  (280.54 - 285.99) | 177.33  (176.66 - 178.00) | 232.66  (229.30 - 236.03) |
| 2001 | 136.79  (128.22 - 145.37) | 217.53  (212.94 - 222.12) | 283.41  (280.70 - 286.12) | 171.41  (170.75 - 172.07) | 234.34  (231.07 - 237.62) |
| 2002 | 140.30  (131.78 - 148.82) | 209.22  (204.85 - 213.59) | 276.12  (273.46 - 278.77) | 167.20  (166.56 - 167.85) | 221.33  (218.22 - 224.44) |
| 2003 | 144.65  (136.01 - 153.28) | 203.16  (198.98 - 207.33) | 268.11  (265.51 - 270.70) | 160.19  (159.56 - 160.81) | 216.31  (213.32 - 219.30) |
| 2004 | 138.12  (129.83 - 146.40) | 191.27  (187.34 - 195.19) | 264.29  (261.74 - 266.84) | 152.48  (151.87 - 153.09) | 206.51  (203.66 - 209.35) |
| 2005 | 140.73  (132.53 - 148.94) | 184.44  (180.73 - 188.14) | 259.01  (256.51 - 261.50) | 149.95  (149.35 - 150.55) | 205.25  (202.50 - 208.01) |
| 2006 | 143.57  (135.37 - 151.78) | 177.99  (174.46 - 181.53) | 252.18  (249.75 - 254.61) | 144.72  (144.13 - 145.31) | 197.56  (194.93 - 200.19) |
| 2007 | 153.69  (145.31 - 162.06) | 169.48  (166.15 - 172.82) | 249.37  (246.98 - 251.76) | 141.43  (140.85 - 142.01) | 193.33  (190.80 - 195.86) |
| 2008 | 139.38  (131.66 - 147.10) | 168.51  (165.28 - 171.74) | 240.14  (237.83 - 242.45) | 141.59  (141.01 - 142.16) | 186.07  (183.66 - 188.48) |
| 2009 | 133.28  (125.90 - 140.65) | 160.30  (157.24 - 163.36) | 232.61  (230.37 - 234.86) | 137.91  (137.34 - 138.47) | 177.26  (174.97 - 179.54) |
| 2010 | 150.45  (142.62 - 158.29) | 158.27  (155.29 - 161.25) | 223.55  (221.37 - 225.73) | 136.13  (135.57 - 136.68) | 177.02  (174.78 - 179.25) |
| 2011 | 140.58  (133.32 - 147.84) | 143.85  (141.13 - 146.56) | 212.85  (210.76 - 214.94) | 133.22  (132.67 - 133.76) | 169.96  (167.86 - 172.07) |
| 2012 | 141.46  (134.36 - 148.56) | 140.60  (138.01 - 143.18) | 210.20  (208.16 - 212.24) | 131.90  (131.37 - 132.44) | 166.46  (164.44 - 168.48) |
| 2013 | 146.03  (139.04 - 153.02) | 137.99  (135.52 - 140.46) | 210.41  (208.41 - 212.42) | 132.67  (132.13 - 133.20) | 164.44  (162.49 - 166.38) |
| 2014 | 147.00  (140.19 - 153.82) | 128.94  (126.64 - 131.23) | 206.27  (204.32 - 208.22) | 130.47  (129.94 - 131.00) | 158.62  (156.77 - 160.47) |
| 2015 | 156.30  (149.44 - 163.17) | 129.57  (127.35 - 131.79) | 206.93  (205.01 - 208.85) | 132.82  (132.29 - 133.35) | 156.38  (154.60 - 158.16) |
| 2016 | 154.40  (147.74 - 161.05) | 124.83  (122.71 - 126.95) | 202.39  (200.52 - 204.26) | 130.52  (130.00 - 131.05) | 155.14  (153.41 - 156.87) |
| 2017 | 154.27  (147.79 - 160.75) | 127.14  (125.07 - 129.21) | 205.95  (204.09 - 207.80) | 132.16  (131.64 - 132.69) | 156.27  (154.58 - 157.96) |
| 2018 | 144.01  (137.94 - 150.07) | 122.17  (120.19 - 124.15) | 204.61  (202.79 - 206.43) | 128.52  (128.01 - 129.04) | 149.75  (148.14 - 151.37) |
| 2019 | 135.36  (129.56 - 141.16) | 113.87  (112.01 - 115.73) | 196.82  (195.06 - 198.58) | 123.71  (123.21 - 124.21) | 141.98  (140.45 - 143.52) |
| 2020 | 173.52  (167.13 - 179.91) | 145.05  (143.01 - 147.09) | 241.51  (239.59 - 243.43) | 137.25  (136.73 - 137.78) | 194.27  (192.53 - 196.02) |
| 2021 | 38.80  (33.52 - 44.08) | 67.34  (56.97 - 77.70) | 71.94  (66.56 - 77.31) | 205.07  (203.20 - 206.93) | 194.34  (192.59 - 196.09) |
| 2022 | 33.15  (28.41 - 37.90) | 61.90  (52.32 - 71.49) | 68.96  (63.90 - 74.01) | 171.48  (169.80 - 173.15) | 162.89  (161.32 - 164.46) |
| 2023 | 34.65  (29.78 - 39.52) | 44.00  (35.96 - 52.03) | 73.10  (67.91 - 78.29) | 151.97  (150.41 - 153.53) | 144.84  (143.37 - 146.30) |
| **Total** | 147.50  (140.08-154.93) | 159.91  (156.92-162.91) | 235.18  (233.00-237.37) | 143.2156  (142.65-143.78) | 184.38  (182.10-186.65) |
| **NH: Non-Hispanic** | | | | | |

**Supplemental Table 4.** Race/ Ethnicity stratified sudden Cardiac arrest-related age-adjusted mortality rates per 100,000 among adults aged 25 and above in the United States, 1999 to 2023.

| **Age-Adjusted Rate Per 100,000 (95% CI)** | | | |
| --- | --- | --- | --- |
| **Year** | **young** | **middle** | **old** |
| 1999 | 12.05 (11.82 - 12.29) | 84.53 (83.79 - 85.26) | 826.05 (823.01 - 829.09) |
| 2000 | 10.24 (10.03 - 10.46) | 81.13 (80.42 - 81.84) | 805.72 (802.74 - 808.70) |
| 2001 | 10.25 (10.04 - 10.47) | 78.89 (78.20 - 79.58) | 784.88 (781.95 - 787.80) |
| 2002 | 10.25 (10.04 - 10.47) | 76.24 (75.58 - 76.90) | 764.48 (761.61 - 767.35) |
| 2003 | 10.29 (10.07 - 10.51) | 74.16 (73.52 - 74.80) | 733.72 (730.93 - 736.51) |
| 2004 | 11.09 (10.86 - 11.32) | 72.90 (72.28 - 73.53) | 696.83 (694.12 - 699.53) |
| 2005 | 11.08 (10.85 - 11.31) | 72.37 (71.76 - 72.98) | 684.86 (682.20 - 687.52) |
| 2006 | 11.06 (10.83 - 11.29) | 71.24 (70.65 - 71.84) | 659.09 (656.51 - 661.68) |
| 2007 | 10.59 (10.36 - 10.81) | 70.00 (69.41 - 70.58) | 645.61 (643.08 - 648.14) |
| 2008 | 10.43 (10.20 - 10.65) | 70.47 (69.89 - 71.05) | 640.13 (637.63 - 642.63) |
| 2009 | 10.64 (10.42 - 10.87) | 70.43 (69.85 - 71.00) | 617.18 (614.74 - 619.61) |
| 2010 | 9.99 (9.77 - 10.21) | 68.66 (68.10 - 69.22) | 610.64 (608.24 - 613.04) |
| 2011 | 9.94 (9.72 - 10.17) | 68.23 (67.68 - 68.78) | 590.75 (588.42 - 593.08) |
| 2012 | 10.14 (9.91 - 10.36) | 68.36 (67.81 - 68.92) | 582.22 (579.93 - 584.50) |
| 2013 | 10.47 (10.24 - 10.70) | 70.58 (70.02 - 71.14) | 580.55 (578.30 - 582.81) |
| 2014 | 10.88 (10.65 - 11.11) | 71.33 (70.77 - 71.88) | 564.63 (562.44 - 566.83) |
| 2015 | 10.82 (10.59 - 11.05) | 73.10 (72.53 - 73.66) | 572.18 (570.00 - 574.36) |
| 2016 | 11.19 (10.96 - 11.42) | 72.87 (72.31 - 73.43) | 558.96 (556.83 - 561.09) |
| 2017 | 11.38 (11.15 - 11.61) | 74.65 (74.08 - 75.22) | 565.45 (563.34 - 567.57) |
| 2018 | 11.45 (11.22 - 11.69) | 74.52 (73.95 - 75.09) | 546.77 (544.72 - 548.82) |
| 2019 | 11.34 (11.10 - 11.57) | 72.72 (72.15 - 73.28) | 522.49 (520.51 - 524.46) |
| 2020 | 15.08 (14.81 - 15.35) | 89.10 (88.48 - 89.73) | 596.62 (594.53 - 598.71) |
| 2021 | 18.20 (17.91 - 18.49) | 98.17 (97.51 - 98.83) | 618.50 (616.32 - 620.68) |
| 2022 | 14.76 (14.50 - 15.02) | 82.96 (82.35 - 83.57) | 563.41 (561.40 - 565.43) |
| 2023 | 12.90 (12.65 - 13.14) | 72.20 (71.63 - 72.77) | 515.79 (513.87 - 517.71) |
| **Total** | 11.46  (11.22-11.69) | 75.19  (74.59-75.80) | 633.90  (631.49-636.31) |

**Supplemental Table 5.** Age group stratified sudden Cardiac arrest-related age adjusted mortality rates per 100,000 among adults aged 25 and above in the United States, 1999 to 2023.

| **Age-Adjusted Rate /100,000 (95% CI)** | | |
| --- | --- | --- |
| **Year** | **Metropolitan** | **Non-Metropolitan** |
| 1999 | 203.26  (202.52 - 204.00) | 166.42  (165.03 - 167.81) |
| 2000 | 196.88  (196.15 - 197.60) | 162.08  (160.72 - 163.44) |
| 2001 | 191.81  (191.11 - 192.52) | 157.88  (156.54 - 159.22) |
| 2002 | 186.44  (185.75 - 187.13) | 154.86  (153.54 - 156.18) |
| 2003 | 179.45  (178.78 - 180.13) | 149.14  (147.85 - 150.43) |
| 2004 | 171.75  (171.10 - 172.40) | 143.42  (142.16 - 144.68) |
| 2005 | 168.94  (168.30 - 169.58) | 141.97  (140.72 - 143.21) |
| 2006 | 163.57  (162.94 - 164.19) | 136.03  (134.82 - 137.25) |
| 2007 | 159.77  (159.16 - 160.38) | 134.94  (133.74 - 136.14) |
| 2008 | 158.20  (157.60 - 158.80) | 136.49  (135.29 - 137.69) |
| 2009 | 153.02  (152.43 - 153.61) | 135.32  (134.12 - 136.51) |
| 2010 | 150.44  (149.87 - 151.02) | 135.10  (133.92 - 136.29) |
| 2011 | 145.79  (145.23 - 146.35) | 133.70  (132.53 - 134.88) |
| 2012 | 143.98  (143.44 - 144.53) | 133.65  (132.48 - 134.82) |
| 2013 | 144.51  (143.97 - 145.06) | 134.38  (133.21 - 135.54) |
| 2014 | 141.22  (140.69 - 141.76) | 134.66  (133.50 - 135.82) |
| 2015 | 143.21  (142.68 - 143.74) | 136.94  (135.77 - 138.10) |
| 2016 | 140.46  (139.94 - 140.98) | 135.81  (134.65 - 136.96) |
| 2017 | 142.33  (141.82 - 142.85) | 138.07  (136.91 - 139.23) |
| 2018 | 138.47  (137.96 - 138.97) | 135.57  (134.43 - 136.71) |
| 2019 | 133.00  (132.51 - 133.49) | 130.24  (129.13 - 131.36) |
| 2020 | 155.66  (155.14 - 156.19) | 148.46  (147.27 - 149.66) |
| **Total** | 159.64  (159.05-160.24) | 141.60  (140.38-142.82) |

**Supplemental Table 6.** Urbanization stratified prematre Cardiac arrest-related age-adjusted mortality rates per 100,000 among adults aged 25 and above in the United States, 1999 to 2020.

| **Age-Adjusted Rate /100,000 (95% CI)** | | | | |
| --- | --- | --- | --- | --- |
| **Year** | **Northeast** | **Midwest** | **South** | **West** |
| 1999 | 280.24  (278.54 - 281.93) | 103.05  (102.08 - 104.01) | 187.51  (186.42 - 188.59) | 234.79  (233.17 - 236.40) |
| 2000 | 273.92  (272.25 - 275.58) | 98.82  (97.87 - 99.76) | 179.23  (178.18 - 180.28) | 230.17  (228.58 - 231.75) |
| 2001 | 267.63  (265.99 - 269.27) | 95.52  (94.59 - 96.44) | 171.29  (170.27 - 172.31) | 229.64  (228.08 - 231.20) |
| 2002 | 264.05  (262.43 - 265.67) | 92.57  (91.67 - 93.48) | 163.95  (162.96 - 164.94) | 224.83  (223.30 - 226.36) |
| 2003 | 255.97  (254.39 - 257.55) | 87.07  (86.20 - 87.94) | 155.91  (154.95 - 156.86) | 220.07  (218.57 - 221.56) |
| 2004 | 248.74  (247.18 - 250.29) | 80.67  (79.83 - 81.50) | 149.35  (148.42 - 150.28) | 210.29  (208.84 - 211.73) |
| 2005 | 248.09  (246.54 - 249.63) | 78.93  (78.11 - 79.75) | 147.00  (146.09 - 147.91) | 204.21  (202.81 - 205.61) |
| 2006 | 238.68  (237.17 - 240.19) | 74.45  (73.66 - 75.24) | 142.26  (141.38 - 143.15) | 200.44  (199.07 - 201.81) |
| 2007 | 235.87  (234.39 - 237.36) | 71.63  (70.86 - 72.40) | 140.19  (139.32 - 141.05) | 193.94  (192.60 - 195.27) |
| 2008 | 230.97  (229.51 - 232.44) | 74.72  (73.94 - 75.50) | 138.49  (137.64 - 139.34) | 193.07  (191.76 - 194.38) |
| 2009 | 220.23  (218.81 - 221.66) | 73.15  (72.38 - 73.91) | 136.23  (135.39 - 137.07) | 187.49  (186.21 - 188.77) |
| 2010 | 209.97  (208.59 - 211.35) | 74.40  (73.63 - 75.17) | 133.47  (132.64 - 134.29) | 190.26  (188.98 - 191.53) |
| 2011 | 201.27  (199.92 - 202.61) | 75.23  (74.46 - 76.00) | 127.26  (126.47 - 128.05) | 188.50  (187.26 - 189.75) |
| 2012 | 195.73  (194.42 - 197.05) | 76.41  (75.64 - 77.18) | 126.29  (125.51 - 127.06) | 186.56  (185.33 - 187.78) |
| 2013 | 194.62  (193.32 - 195.92) | 79.90  (79.12 - 80.68) | 125.98  (125.21 - 126.74) | 187.10  (185.89 - 188.30) |
| 2014 | 190.65  (189.37 - 191.93) | 81.19  (80.41 - 81.97) | 124.40  (123.65 - 125.15) | 179.29  (178.13 - 180.46) |
| 2015 | 194.51  (193.23 - 195.80) | 83.27  (82.49 - 84.06) | 123.60  (122.86 - 124.34) | 184.16  (183.00 - 185.32) |
| 2016 | 190.20  (188.93 - 191.47) | 83.79  (83.01 - 84.58) | 120.91  (120.19 - 121.64) | 180.54  (179.40 - 181.68) |
| 2017 | 187.05  (185.81 - 188.29) | 87.08  (86.28 - 87.87) | 122.88  (122.16 - 123.60) | 185.37  (184.23 - 186.51) |
| 2018 | 181.31  (180.10 - 182.52) | 88.99  (88.19 - 89.78) | 119.07  (118.37 - 119.76) | 178.42  (177.32 - 179.53) |
| 2019 | 175.27  (174.09 - 176.45) | 90.08  (89.29 - 90.88) | 110.12  (109.45 - 110.78) | 173.16  (172.08 - 174.23) |
| 2020 | 212.38  (211.08 - 213.68) | 101.46  (100.61 - 102.30) | 125.38  (124.68 - 126.08) | 203.61  (202.46 - 204.76) |
| 2021 | 202.51  (201.23 - 203.80) | 105.58  (104.70 - 106.46) | 141.09  (140.33 - 141.85) | 219.99  (218.76 - 221.22) |
| 2022 | 187.84  (186.62 - 189.05) | 97.41  (96.59 - 98.24) | 119.17  (118.48 - 119.85) | 198.86  (197.72 - 200.00) |
| 2023 | 170.88  (169.72 - 172.03) | 88.92  (88.13 - 89.71) | 102.20  (101.57 - 102.82) | 187.51  (186.41 - 188.62) |
| **Total** | 218.3432  (216.95-219.74) | 85.7716  (84.95-86.59) | 137.33  (136.50-138.15) | 198.89  (197.60-200.18) |

**Supplemental Table 7.** Region-stratified sudden Cardiac arrest-related age-adjusted mortality rates per 100,000 among adults aged 25 and above in the United States, 1999 to 2023.

| **State** | **Age-Adjusted Rate /100,000 (95% CI)** | **Percentile** |
| --- | --- | --- |
| Alabama | 209.24 (208.19 - 210.29) | 87.23% |
| Alaska | 67.38 (65.30 - 69.46) | 18.37% |
| Arizona | 124.98 (124.29 - 125.68) | 63.27% |
| Arkansas | 135.18 (134.12 - 136.24) | 67.35% |
| California | 285.74 (285.28 - 286.21) | 93.88% |
| Colorado | 73.04 (72.38 - 73.70) | 28.57% |
| Connecticut | 258.24 (256.98 - 259.51) | 85.71% |
| Delaware | 49.95 (48.80 - 51.10) | 12.24% |
| District of Columbia | 81.31 (79.38 - 83.23) | 36.73% |
| Florida | 144.63 (144.24 - 145.02) | 79.59% |
| Georgia | 293.67 (292.69 - 294.65) | 95.92% |
| Hawaii | 213.99 (212.11 - 215.88) | 85.71% |
| Idaho | 71.74 (70.62 - 72.85) | 26.53% |
| Illinois | 59.60 (59.26 - 59.95) | 6.12% |
| Indiana | 84.27 (83.70 - 84.84) | 38.78% |
| Iowa | 79.22 (78.47 - 79.97) | 32.65% |
| Kansas | 119.56 (118.56 - 120.57) | 61.22% |
| Kentucky | 119.52 (118.68 - 120.36) | 59.18% |
| Louisiana | 90.24 (89.51 - 90.97) | 42.86% |
| Maine | 62.97 (61.97 - 63.97) | 14.29% |
| Maryland | 36.28 (35.88 - 36.69) | 2.04% |
| Massachusetts | 157.35 (156.61 - 158.09) | 77.55% |
| Michigan | 69.67 (69.26 - 70.08) | 24.49% |
| Minnesota | 35.29 (34.88 - 35.70) | 0.00% |
| Mississippi | 293.44 (291.82 - 295.06) | 97.96% |
| Missouri | 92.02 (91.42 - 92.63) | 44.90% |
| Montana | 69.30 (68.04 - 70.57) | 22.45% |
| Nebraska | 197.06 (195.47 - 198.65) | 81.63% |
| Nevada | 272.66 (270.91 - 274.40) | 91.84% |
| New Hampshire | 89.97 (88.69 - 91.24) | 40.82% |
| New Jersey | 134.45 (133.85 - 135.05) | 69.39% |
| New Mexico | 90.18 (89.10 - 91.25) | 46.94% |
| New York | 384.82 (384.14 - 385.51) | 100.00% |
| North Carolina | 105.88 (105.34 - 106.42) | 55.10% |
| North Dakota | 96.84 (95.06 - 98.63) | 51.02% |
| Ohio | 136.44 (135.92 - 136.97) | 65.31% |
| Oklahoma | 136.30 (135.35 - 137.26) | 63.27% |
| Oregon | 46.71 (46.17 - 47.24) | 10.20% |
| Pennsylvania | 112.75 (112.32 - 113.19) | 57.14% |
| Rhode Island | 142.77 (141.07 - 144.48) | 73.47% |
| South Carolina | 139.80 (138.92 - 140.68) | 71.43% |
| South Dakota | 68.69 (67.29 - 70.08) | 20.41% |
| Tennessee | 132.67 (131.94 - 133.40) | 69.39% |
| Texas | 100.38 (100.03 - 100.74) | 16.33% |
| Utah | 79.68 (78.66 - 80.71) | 34.69% |
| Vermont | 57.53 (56.09 - 58.98) | 8.16% |
| Virginia | 95.77 (95.20 - 96.34) | 49.00% |
| Washington | 76.91 (76.36 - 77.45) | 30.61% |
| West Virginia | 169.89 (168.48 - 171.30) | 75.51% |
| Wisconsin | 36.87 (36.48 - 37.27) | 4.08% |
| Wyoming | 63.08 (61.34 - 64.82) | 12.24% |

**Supplemental Table 8.** State-stratified sudden cardiac arrest-related age-adjusted mortality rates per 100,000 and their respective percentiles among adults aged 25 and above in the United States, 1999 to 2020.
